# Oxidative Stress and Dynamic Thiol/Disulfide Homeostasis in Autism: A Focus on Early Childhood

**DOI:** 10.1007/s12031-025-02358-z

**Published:** 2025-05-02

**Authors:** Halenur Teke, Senay Balci, Salim Neselioglu, Selçuk Teke, Ozcan Erel, Lulufer Tamer, Fevziye Toros

**Affiliations:** 1 Department of Child and Adolescent Psychiatry, Medical Faculty, Mersin University, Ankara, Turkey; 2 Department of Medical Biochemistry, Medical Faculty, Mersin University, Mersin, Turkey; 3 Department of Biochemistry, Ankara Bilkent City Hospital, Yıldırım Beyazit University, Ankara, Turkey; 4 Department of Pediatrics, Medical Faculty, Mersin University, Ankara, Turkey; 5 Department of Child and Adolescent Psychiatry, Medical Faculty, Mersin University, Mersin, Turkey

**Keywords:** Autism spectrum disorder, Oxidative stress, Dynamic thiol/disulfide homeostasis, Biomarkers, Early childhood

## Abstract

Autism spectrum disorder (ASD) is a complex neurodevelopmental condition with multifactorial etiopathogenesis, where oxidative stress (OS) has been implicated as a key contributing factor. This study aimed to evaluate the plasma dynamic thiol/disulfide homeostasis (DTDH) parameters—a relatively novel OS biomarker—alongside classical OS biomarkers, including total oxidant status (TOS), total antioxidant status (TAS), oxidative stress index (OSI), glutathione, and glutathione peroxidase (GPx), in preschool children diagnosed with ASD. A total of 49 children with ASD and 31 age- and sex-matched typically developing children between the ages of 2 and 6 years were included. In addition to sociodemographic data collection, the Childhood Autism Rating Scale (CARS) and Clinical Global Impression-Severity Scale (CGI-S) were administered to assess autism severity. Blood samples were analyzed using automated spectrophotometric techniques to determine OS biomarkers. The results demonstrated that DTDH parameters and classical OS markers exhibited parallel changes; however, no statistically significant differences were detected between the ASD and control groups across all OS markers. Furthermore, no significant association was found between OS biomarkers and autism severity. Moreover, we intentionally restricted our sample to a younger age group to enable a focused examination of OS dynamics during early developmental stages. This study underscores the potential impact of age as a critical determinant in OS-related alterations in autism and highlights the need for further age-stratified investigations to elucidate the role of OS in ASD pathophysiology and its potential diagnostic relevance.

## Introduction

Autism spectrum disorder (ASD) is a multifaceted neurodevelopmental condition that manifests as impairments in social interaction, challenges in verbal and nonverbal communication, and a tendency toward repetitive behaviors and narrowly focused interests (American Psychiatric Association 2013). The prevalance of ASD has shown a rising trend over the years; as of 2023, according to the CDC report in the USA, the prevalence of ASD has been determined as 1/36 (approximately 2.8%) (Maenner et al. 2023). Although the exact cause remains unclear, ASD is believed to arise from complex interactions between genetic, immune, and environmental factors, with oxidative stress (OS) emerging as a crucial contributor to its pathophysiology (Pangrazzi et al. 2020).

OS occurs when the generation of reactive oxygen species (ROS, also known as free radicals) exceeds the neutralizing capacity of antioxidant defense systems, leading to protein and DNA oxidation, as well as lipid peroxidation in cells, which ultimately causes degeneration (Schieber and Chandel 2014). The central nervous system is particularly susceptible to OS due to its high oxygen utilization, significant lipid content, and limited antioxidant defenses (Hulbert et al. 2007). Research suggests that OS appears to be important in the etiopathogenesis of neuropsychiatric disorders by participating in the underlying biochemical mechanisms (Salim 2017). Recently, OS has gained increasing recognition as a key factor in the pathogenesis of ASD. Studies have shown damage in different regions of the brain in children with ASD, potentially caused by OS (Usui et al. 2023). Several studies have investigated various biomarkers related to OS in blood, urine, and post-mortem brain tissue, with most findings indicating an impaired oxidant-antioxidant balance in individuals with ASD (Liu et al. 2022; Osredkar et al. 2019; Rose et al. 2012). In addition, genetic predisposition, environmental factors and mitochondrial dysfunction are thought to play a role in oxidative imbalance, affecting early central nervous system development and exacerbating ASD symptoms (Balachandar et al. 2021).

The antioxidant defense system is essential for preserving cellular integrity by neutralizing the OS. It consists of two main components: enzyme-based and non-enzyme-based antioxidants. These enzymatic systems (e.g., glutathione peroxidase (Gpx), superoxide dismutase) facilitate the degradation of ROS, thereby limiting cellular damage. In parallel, non-enzyme-based antioxidants such as beta-carotene, vitamin C, glutathione and other thiol-containing compounds directly neutralize free radicals. Together, these mechanisms work synergistically to maintain cellular homeostasis and prevent the development of OS-related pathologies (Francenia Santos-Sánchez et al. 2019).

Numerous studies and meta-analyses have shown that an imbalance in glutathione-dependent redox metabolism is frequently observed in individuals with ASD (Chen et al. 2021; Bjørklund et al. 2021, 2020). Glutathione, a non-protein thiol substance, is essential for the neutralization of ROS (Aoyama 2021). Several studies have consistently reported lower blood glutathione levels in children with ASD than in healthy controls (Nasrallah and Alzeer. 2022; Castejon et al. 2021). Moreover, findings from post-mortem studies exploring the involvement of OS in ASD pathophysiology indicate that autism is linked to reduced glutathione-based antioxidant defense in specific brain regions, with the cerebellum and temporal lobe being particularly affected (Chauhan et al. 2012, Rose et al. 2012). A meta-analysis published in 2021 proposed that the consistent and large effect size of glutathione metabolism biomarkers suggests their potential to aid in the early diagnosis of ASD (Chen et al. 2021).

GPx is one of the primary antioxidant enzymes that convert hydrogen peroxide to water using reduced glutathione. Several studies have identified reduced GPx activity in ASD cases, contributing to the ineffective neutralization of OS (Nadeem et al. 2019; Gu et al 2013; Ghanizadeh et al. 2012).

Thiols are a class of organic molecules characterized by the presence of sulphhydryl (-SH) groups, which are essential for cellular defense against OS. Plasma thiols primarily comprise albumin- and protein-bound thiols (R-SH), while a lesser proportion consists of low molecular weight thiols (e.g., cysteine and glutathione) (Turell et al. 2013). ROS promote the oxidation of thiol groups, which leads to the formation of reversible disulfide bonds. They can be reduced, allowing the regeneration of thiol groups and the maintenance of thiol/disulfide homeostasis (Jones and Liang 2009). Dynamic thiol/disulfide homeostasis (DTDH) is essential for maintaining cellular balance, contributing to antioxidant defense, programmed cell death, enzymatic activity, gene expression regulation, and intracellular signaling (Eren et al. 2015; Circu and Aw 2010). Plasma concentrations of thiol/disulfide pairs are considered to be reliable and valuable biomarkers of in vivo OS and antioxidant defenses (Pérez et al. 2012). Growing evidence suggests that aberrant DTDHs play a role in the pathogenesis of a wide range of disorders, including diabetes mellitus, cardiovascular pathologies, neoplastic conditions, inflammatory arthritis, Parkinsonian syndromes, Alzheimer-type dementia, and demyelinating diseases (Erel and Neselioglu 2014; Erel and Erdoğan 2020).

For many years, various methods have been used to analyze thiol levels. However, previous research has predominantly focused on low molecular weight thiols, such as reduced and oxidized glutathione, as biomarkers of OS in ASD (Głowacki and Bald 2009). Consequently, thiol and disulfide levels determined using traditional measurement techniques may not provide an accurate representation of the body’s thiol/disulfide balance. In recent years, an innovative automated technique introduced by Erel and Neselioğlu (2014) has been utilized for assessing thiol/disulfide levels. This technique enables the quantification of both native thiol and disulfide concentrations, where their combined total is defined as total Thiol. Several studies utilizing this novel method have investigated DTDH in children with Obsessive–Compulsive Disorder (Ozkan et al. 2021), Attention Deficit Hyperactivity Disorder (Avcil et al. 2017), and ASD (Efe et al. 2021; Ayaydın et al. 2021), consistently indicating a predominance of OS.

Efforts to identify biomedical treatments targeting the core symptoms of ASD have, so far, been largely unsuccessful (Persico et al. 2021). However, early intervention in children with ASD has been shown to improve their social skills later in life (Park et al. 2023). The relationship between OS and ASD may offer valuable insights into the underlying pathophysiological mechanisms of the disorder, assist in identifying biomarkers for early diagnosis, and aid in discovering potential therapeutic targets. Consequently, new OS markers are being explored in ASD patients, with some gaining scientific recognition (Liu et al. 2022). Among these, DTDH parameters have recently been highlighted as key biomarkers linking autism and OS (Ayaydın et al. 2021; Efe et al. 2021). However, comparing newly recognized OS biomarkers with classical parameters is essential to better understand this relationship and determine which biomarkers are more specific and sensitive in ASD (Chen et al. 2021). Current research underscores the necessity for further investigations in this field, particularly focusing on heterogeneous ASD populations across different age groups (Chen et al. 2021; Frustaci et al. 2012). To the best of our knowledge, no study has assessed DTDH parameters in children with autism in comparison to classical parameters, and OS parameters have not been specifically examined in young children with autism.

The aim of this study was to investigate the oxidant-antioxidant balance in ASD by analyzing DTDH, total oxidant status (TOS), total antioxidant status (TAS), oxidative stress index (OSI), glutathione and GPx levels in the peripheral blood of preschool children (aged 2–6 years) with autism.

## Materials and Methods

### Participants and Process

Forty‐nine children (39 boys and 10 girls) aged between 2 and 6 years were enrolled in the study group; these participants were either newly diagnosed with ASD or were under follow‐up at the Mersin University Faculty of Medicine Child and Adolescent Psychiatry Outpatient Clinic. The control group consisted of 31 age- and sex-matched children (26 boys and 5 girls) recruited from the pediatric outpatient clinic of our hospital and were released without any psychopathological diagnosis or intervention. Prior to data collection, a formal power analysis was performed based on effect sizes reported in previous studies examining oxidative stress biomarkers in children with ASD. The analysis indicated that a minimum of 30 participants per group would be sufficient to detect statistically significant differences with adequate power. Accordingly, we aimed to recruit at least 30 children in each group, and ultimately included 49 children with ASD and 31 typically developing children.

Since the study population consisted of preschool-aged children, formal intelligence testing was not conducted. Instead, a trained clinical psychologist administered the Denver Developmental Screening Test to both groups to assess general developmental status. For both groups, participants were excluded if they presented with any coexisting conditions involving severe global developmental delay, genetic syndromes, or chronic medical conditions (e.g., neurological, cardiac, autoimmune, endocrine, neoplastic, hematological, infectious, or metabolic disturbances). In addition, children receiving antioxidants (such as vitamin C), following a special diet, or taking supplements that could alter OS resistance were not eligible. Exclusion criteria also encompassed any current or past medication use within the preceding year, as well as a history of severe head trauma. A pre-constructed questionnaire was administered to both the ASD and control cohorts to collect a range of socio-demographic and clinical data. In addition, the medical records of all participants were thoroughly examined. The diagnosis of ASD was established following a comprehensive clinical interview, adhering to the diagnostic framework described in the fifth edition of the Diagnostic and Statistical Manual of Mental Disorders (DSM-5). The Turkish versions of the Childhood Autism Rating Scale (CARS) and the Clinical Global Impression-Severity Scale (CGI-S) were used to both the ASD and control groups. The CARS, which comprises 15 items, is a tool that has been designed to evaluate the severity of autism in children. Scores below 30 suggest the absence of significant features of autism. Scores ranging from 30 to 36.5 are classified as indicative of mild-to-moderate autism, whereas scores from 37 to 60 are considered representative of severe autism. The CGI-S Scale is a seven-point scale that is used by clinicians for the purpose of assessing the severity of a patient’s condition at the time of their evaluation. This is determined by the clinician's prior experience with patients presenting with an identical diagnosis.

Forty‐nine children (39 boys and 10 girls) aged between 2 and 6 years were enrolled in the study group; these participants were either newly diagnosed with ASD or were under follow‐up at the Mersin University Faculty of Medicine Child and Adolescent Psychiatry Outpatient Clinic. The control group consisted of 31 age- and sex-matched children (26 boys and 5 girls) recruited from the pediatric outpatient clinic of our hospital and were released without any psychopathological diagnosis or intervention. Prior to data collection, a formal power analysis was performed based on effect sizes reported in previous studies examining oxidative stress biomarkers in children with ASD. The analysis indicated that a minimum of 30 participants per group would be sufficient to detect statistically significant differences with adequate power. Accordingly, we aimed to recruit at least 30 children in each group, and ultimately included 49 children with ASD and 31 typically developing children. For both groups, participants were excluded if they presented with any coexisting conditions involving genetic, neurological, cardiac, autoimmune, endocrine, neoplastic, hematological, infectious, or metabolic disturbances. In addition, children receiving antioxidants (such as vitamin C), following a special diet, or taking supplements that could alter OS resistance were not eligible. Exclusion criteria also encompassed any current or past medication use within the preceding year, as well as a history of severe head trauma. A pre-constructed questionnaire was administered to both the ASD and control cohorts to collect a range of socio-demographic and clinical data. In addition, the medical records of all participants were thoroughly examined. The diagnosis of ASD was established following a comprehensive clinical interview, adhering to the diagnostic framework described in the fifth edition of the Diagnostic and Statistical Manual of Mental Disorders (DSM-5). The Turkish versions of the Childhood Autism Rating Scale (CARS) and the Clinical Global Impression-Severity Scale (CGI-S) were used to both the ASD and control groups. The CARS, which comprises 15 items, is a tool that has been designed to evaluate the severity of autism in children. Scores below 30 suggest the absence of significant features of autism. Scores ranging from 30 to 36.5 are classified as indicative of mild-to-moderate autism, whereas scores from 37 to 60 are considered representative of severe autism. The CGI-S Scale is a seven-point scale that is used by clinicians for the purpose of assessing the severity of a patient's condition at the time of their evaluation. This is determined by the clinician’s prior experience with patients presenting with an identical diagnosis.

### Blood Sampling and Processing Procedures

Following a 12-h fasting period, venous blood specimens were drawn from the antecubital vein of both the patient and control groups. The collected blood was placed into plain tubes, and serum was separated by centrifugation at 4000 rpm for 10 min. The resulting serum was then preserved at − 80 °C until the time of analysis. All biochemical evaluations were conducted after the entire set of samples had been obtained. DTDH parameters, TOS, TAS, OSI, glutathione, and GPx were measured in the serum samples.

Serum DTDH parameters were analyzed using a fully automated spectrophotometric technique. In this method, disulfide bonds are reduced to free thiols by sodium borohydride, and the remaining borohydride is neutralized with formaldehyde. Total thiol (ToSH) and native thiol (SH) levels are then measured, with disulfide (SS) concentration calculated as half the difference between them. These values allow the determination of key redox ratios, including the reduced thiol ratio (SH/ToSH), oxidized thiol ratio (SS/ToSH), and thiol oxidation–reduction ratio (SS/SH), providing insights into redox balance. The parameters of DTDH were expressed in mmol/L.

TOS, TAS and OSI were measured using the Erel method (Erel 2005). Blood glutathione levels were measured using the DTNB (Ellman’s reagent) method, while GPx activity was assessed by a spectrophotometric method based on NADPH consumption.

### Ethics

The parents of all participants gave their documented informed consent, following a thorough explanation of the aims and procedures of the study. The study was ethically authorized by the Faculty Ethics Committee and conducted in accordance with the 2013 update of the Helsinki Declaration.

### Funding

This research was supported by the Mersin University Scientific Research Coordination Office (protocol no: 2017–1 TP3-2198).

### Statistical Analysis

All statistical analyses were conducted using SPSS software version 11.5 (IBM SPSS for Windows, Chicago, IL, USA). Descriptive statistics were applied to summarize continuous variables, including blood parameters and scale scores, using mean, standard deviation, median, minimum and maximum values, as well as 25 th and 75 th percentiles. Categorical variables were presented as frequencies and percentages.

The Shapiro–Wilk test was used to assess the normality of data distribution. Differences in means between the ASD and control groups, as well as comparisons across CARS autism severity and CGI severity groups, were analyzed using Student’s *t*-test, while differences in medians were evaluated with the Mann–Whitney *U* test. For categorical variables, including gender, parental education level, parental psychiatric history, total monthly income, and parental consanguinity, comparisons between groups were performed using the chi-square test.

Correlation analysis was conducted to examine the relationships between continuous variables using the Pearson correlation coefficient (r). Additionally, multiple regression analysis was performed to establish predictive models for variables showing significant associations. A *P*-value < 0.05 was considered statistically significant for all analyses.

## Results

### Demographic and Clinical Characteristics of Participants

In the ASD group, 79.6% (*n* = 39) were male, while 20.4% (*n* = 10) were female, closely reflecting the gender distribution in the control group, where 80.6% (*n* = 25) were male and 19.4% (*n* = 6) were female. The mean age in the ASD group was 46.8 ± 17.74 months, compared to 48.4 ± 15.04 months in the control group. Statistical analysis revealed no significant differences between the two groups in terms of mean age (*P* = 0.66) or gender distribution (*P* = 0.909). A comprehensive summary of demographic characteristics for both groups is provided in Table 1.

**Table 1:** Sociodemographic features of children with ASD and the control group

	*ASD group (n* = *49)*	*Control group (n* = *31)*	*P*
*Age (months)*			
*Mean* ± *SD*	46.8 ± 17.74	48.4 ± 15.04	0.66
*Median [min–max]*	42.00 (19–84)	46.00 (21–72)	
*Sex n (%)*			
*Males/females*	39 (79.6%)/10 (20.%)	25 (80.6%)/6 (19.4%)	0.909
*Ages of parents at birth (yrs)*			
*Mean* ± *SD, median [min–max]*			
*Mothers*	28.5 ± 3.58	27.16 ± 2.73	0.07
*Fathers*	31.76 ± 4.39	28.29 ± 3.19	< 0.001
*Parental psychiatric histories n(%)*	12 (24.5%)	3 (9.7%)	0.098
*Consanguinity of parents n (%)*	10 (20.4%)	5 (16.1%)	0.633

When examining parental age at childbirth, no statistically significant difference was observed between groups regarding maternal age (*P* = 0.07). However, paternal age at birth was significantly higher in the ASD group compared to the control group (*P* < 0.01, Table 1). Regression analysis indicated that each additional year of paternal age was associated with a 1.272-fold increase in the risk of autism (*P* = 0.001, 95% CI [1.102–1.468]).

Furthermore, no significant differences were identified between the ASD and control groups concerning maternal (*P* = 0.476) and paternal (*P* = 0.389) educational levels, total monthly income (*P* = 0.438), parental consanguinity (*P* = 0.633), or parental psychiatric history (*P* = 0.098).

Among the 49 children in the ASD group, 22 (44.8%) were classified as having mild to moderate autism, while 27 (55.1%) were categorized as having severe autism based on their CARS scores. Notably, children in the severe autism subgroup were significantly younger than those with mild to moderate autism (*P* = 0.016). According to regression analysis, each additional year of age was associated with a 0.153-point decrease in the CARS score (*r* = − 0.384, *P* = 0.006).

No significant differences were detected between the mild-to-moderate and severe autism subgroups regarding gender distribution (*P* = 0.074), maternal and paternal age at birth (*P* = 0.449 and *P* = 0.232, respectively), parental education level (*P* = 0.584 for both parents), total monthly income (*P* = 0.248), parental consanguinity (*P* = 0.282), or parental psychiatric history (*P* = 0.111).

### Comparison of Groups Based on DTDH, TOS, TAS, OSI, Glutathione, GPx, and Their Correlation with Autism Symptom Severity

There were no significant differences between the ASD and control groups in mean native thiol (*P* = 0.93), total thiol (*P* = 0.37), disulfide (*P* = 0. 69), reduced thiol ratio (*P* = 0.459), oxidised thiol ratio (*P* = 0.459), thiol oxidation–reduction ratio (*P* = 0.459), TOS (*P* = 0.80), TAS (*P* = 0.72), OSI (*P* = 0.719), glutathione (*P* = 0.30) and GPx (*P* = 0.96), as shown in Table 2.

**Table 2:** Comparison of oxidative stress indicators between ASD and control groups

	*ASD group (n* = *49)*	*Control group (n* = *31)*	
	*Mean* ± *SD* *Median [min–max]*	*Mean* ± *SD* *Median [min–max]*	*P*
*Native thiol (SH) (µmol/L)*	468.57 ± 36.05 464.90 (380.7–564.4)	469.19 ± 31.47 467.20 (411.4–540.9)	0.93
*Total thiol (ToSH) (µmol/L)*	513.90 ± 38.95 509.00 (434.2–622.4)	510.66 ± 34.93 502.20 (430.1–588.6)	0.37
*Disulfide (SS) (µmol/L)*	22.67 ± 12.08 21.90 (6.40–55.20)	20.74 ± 11.99 20.50 (1.20–52.80)	0.69
*Redox potential (SS/SH)*	4.90 ± 2.75 4.69 (1.41–12.10)	4.47 ± 2.64 4.10 (0.24–11.29)	0.459
*Reduced thiol ratio (SH/ToSH)*	91.27 ± 4.44 91.43 (80.51–97.25)	91.99 ± 4.31 92.43 (81.57–99.53)	0.459
*Oxidized thiol ratio (SS/ToSH)*	4.36 ± 2.15 3.79 (0.24–9.21)	4.00 ± 2.15 3.79 (0.24–9.21)	0.459
*TAS*	1.73 ± 0.1 1.73 (1.51–1.98)	1.73 ± 0.08 1.76 (1.55–1.86)	0.72
*TOS*	4.64 ± 4.06 3.05 (1.58–19.37)	3.55 ± 1.58 3.07 (1.88–7.46)	0.80
*OSİ*	266.071 ± 223.020 176.07 (89.27–1058.47)	203.682 ± 88.637 177.65 (111.90–431.21)	0.719
*Glutathione (µg/ml)*	23.71 ± 12.03 22.01 (2.20–52.09)	21.00 ± 10.33 21.76 (1.98–51.32)	0.30
*GPx*	1012.80 ± 492.56 937.15 (250.14–1996.30)	1017.43 ± 513.67 823.74 (291.11–1895.98)	0.96

In the ASD group, correlation analysis revealed no linear association between plasma oxidative stress markers and autism symptom severity as assessed by the CGI-S scale and CARS (Table 3).

**Table 3:** Comparison of oxidative stress indicators between mild-to-moderate and severe autism groups according to CARS

	*Mild-to-moderate autism (n* = *22)*	*Severe autism (n* = *27)*	
	*Mean* ± *SD* *Median [min–max]*	*Mean* ± *SD* *Median [min–max]*	*P*
*Native thiol (SH) (µmol/L)*	476.55 ± 38.24 474.90 (422.1–564.4)	462.06 ± 33.46 459.20 (380.7–525.1)	0.16
*Total thiol (ToSH) (µmol/L)*	521.89 ± 41.92 509.55 (460.5–622.4)	507.38 ± 35.81 506.80 (434.2–566.9)	0.19
*Disulfide (SS) (µmol/L)*	22.66 ± 11.81 21.85 (7.10–51.20)	22.66 ± 12.52 21.90 (6.40–55.20)	1.00
*Redox potential (SS/SH)*	4.81 ± 2.65 4.68 (1.46–10.80)	4.98 ± 2.88 5.07 (1.41–12.10)	0.809
*Reduced thiol ratio (SH/ToSH)*	91.40 ± 4.26 91.44 (82.24–97.17)	91.17 ± 4.66 90.80 (80.51–97.25)	0.809
*Oxidized thiol ratio (SS/ToSH)*	4.29 ± 2.13 4.27 (1.42–8.88)	4.41 ± 2.33 4.60 (1.37–9.74)	0.809
*TAS*	1.73 ± 0.08 1.76 (1.55–1.86)	1.73 ± 0.1 1.73 (1.51–1.98)	0.72
*TOS*	3.55 ± 1.58 3.07 (1.88–7.46)	4.64 ± 4.06 3.05 (1.58–19.37)	0.80
*OSİ*	203.682 ± 88.637 177.65 (111.90–431.21)	266.071 ± 223.020 176.07 (89.27–1058.47)	0.719
*Glutathione (µg/ml)*	21.00 ± 10.33 21.76 (1.98–51.32)	23.71 ± 12.03 22.01 (2.20–52.09)	0.30
*GPx*	1017.43 ± 513.67 823.74 (291.11–1895.98)	1012.80 ± 492.56 937.15 (250.14–1996.3)	0.96

## Discussion

In the current study, we investigated DTDH and classical OS biomarkers in preschool children with ASD, a population that remains underrepresented in OS research. Contrary to most previous findings, our results did not reveal any statistically significant differences between the ASD and control groups in terms of DTDH parameters, including mean native thiol, total thiol, disulfide, reduced thiol ratio, oxidized thiol ratio, and thiol oxidation–reduction ratio, as well as TOS, TAS, OSI, glutathione, or GPx levels. Moreover, we did not observe any significant correlations between OS biomarkers and the severity of autism symptoms as measured by CARS and CGI-S scores. Although paternal age was significantly higher in the ASD group compared to controls, no significant correlations were found between paternal age and either OS parameters or autism severity scores within the ASD group. While advanced paternal age has been associated with an increased risk of ASD onset, current evidence does not clearly support a direct relationship between paternal age and OS levels or symptom severity (Grice et al. 2017). Our findings are consistent with this perspective, as no significant associations were observed in our sample.

Several previous studies have consistently reported increased OS and impaired antioxidant defense systems in individuals with ASD, suggesting OS as a key factor in ASD pathophysiology (Usui et al. 2023; Liu et al. 2022; Pangrazzi et al. 2020). In particular, altered glutathione metabolism and reduced GPx activity have been implicated in both peripheral and central nervous system changes (Chen et al. 2021; Nadeem et al. 2019; Rose et al. 2012). More recently, DTDH has emerged as a novel and sensitive biomarker reflecting redox imbalance in psychiatric and neurodevelopmental disorders, including ASD (Tuncay et al. 2023). However, our findings did not align with these commonly reported alterations, which may, in part, be explained by the younger age of our sample compared to those in previous studies.

We identified two cross-sectional studies that investigated DTDH parameters in children with ASD (Ayaydın et al. 2021; Efe et al. 2021). Unlike our results, both studies reported significant shifts in thiol/disulfide balance indicative of increased OS in children with autism. The mean ages in their ASD samples were above 70 months, whereas our study focused on a younger group (mean age 46.8 months). This age discrepancy may partly account for the absence of significant redox imbalance in our cohort and highlights the potential role of age in modulating OS levels in ASD.

While the younger age range of our cohort compared to other studies may contribute to the lack of significant group differences, this factor alone may not fully explain the null findings. Other unmeasured variables—such as inter-individual biological variability, differences in ASD subtypes, early-stage ASD pathophysiology and oxidative changes, or even unmeasured environmental influences—could also have played a role. Additionally, the possibility that OS alterations may emerge more clearly at later developmental stages cannot be excluded.

Although OS has been consistently linked to ASD, its precise role in the etiopathogenesis of the disorder remains unclear (González-Fraguela 2013). Several studies suggest that OS-related changes are influenced by various factors, including age, disease severity, diet, and environmental exposures (Frustaci et al. 2012). The absence of a significant oxidative imbalance in the current study indicates that age may play a critical role in modulating biochemical processes. Numerous investigations point to a complex relationship between ASD and immune dysregulation/inflammation, OS, mitochondrial dysfunction, and toxicant exposures (Rossignol and Frye 2012; Usui et al. 2023). While OS may not be pronounced in the early stages of ASD, it could progressively increase due to neuroinflammatory processes, mitochondrial dysfunction, and environmental factors (Usui et al. 2023; Rose et al. 2014; Deth 2008; Chauhan and Chauhan 2015). As we know, antioxidant capacity declines with age, leading to increased OS levels (Pandey and Rizvi 2010; Rizvi and Maurya 2007). In this context, cellular antioxidant defense mechanisms may help maintain oxidative balance during the early development of ASD, potentially preventing a pronounced oxidative imbalance in young children Therefore, when evaluating OS and antioxidant capacity in ASD, it is crucial to consider the individual’s age (Chen et al. 2021).

Limited research has explored the relationship between OS biomarkers and age in children with autism (Morimoto et al. 2020, 2023). In their study investigating OS in ASD, M. Morimoto et al. (2020) divided the subjects into two different age groups: toddlers/preschoolers between 2 and 6 years of age, and school-age children between 7 and 15 years of age. According to the study, reactive oxygen metabolite levels were significantly elevated in children with ASD between 7 and 15 years old compared to their typically developing peers in the same age range. In contrast, no significant differences in plasma levels of reactive oxygen metabolites were observed when comparing typically developing children and children with ASD aged 2 to 6 years. This suggests that OS may increase with age in children with ASD (Morimoto et al. 2020).

In 2021, a systematic review and meta-analysis of blood OS marker profiles in children with ASD was performed by Chen et al. (2021). The results showed that children with ASD had elevated OS markers and reduced antioxidant levels compared to typically developing children. However, no significant differences were seen for 17 potential markers that had previously shown significant differences in many studies. The authors emphasized that there was heterogeneity between studies for a majority of markers and that meta-regression analyses suggested that age and publication year could affect the meta‐analytic results.

Since the diagnosis of autism still primarily relies on behavioral criteria and its presentation varies among individuals, contemporary research has focused on identifying specific biological markers that can be rapidly detected through simple biochemical assays, enabling early risk assessment and timely diagnosis (Liu et al. 2022). Although many studies suggest that OS markers are potentially promising for early diagnosis of ASD, Frustaci et al. (2012, meta-analysis) state that heterogeneous ASD populations should be studied in different age groups to improve the diagnostic accuracy of biomarkers. In the current study, OS markers in young children with autism were similar to those in healthy children, suggesting that these markers may not be suitable for early diagnosis. Therefore, while age appears to be a contributing factor, more focused research is required to determine the exact link between age and OS levels in children with ASD.

The present research has several methodological constraints, and therefore the results should be interpreted with caution. Firstly, only specific OS markers were evaluated, and other relevant biomarkers—particularly those associated with mitochondrial dysfunction or inflammation—were not examined. The generalizability of the findings is limited by several factors, including the relatively small sample size, the cross-sectional design, the single-center setting, and the inclusion of only participants recruited from a hospital-based outpatient clinic. Due to sample size limitations, subgroup analyses within the 2–6 year age range (e.g., comparing 2- vs. 6-year-olds) could not be performed, which restricts the granularity of age-specific interpretations. Moreover, the exclusion of children under the age of two—due to diagnostic challenges in this age group—further limits the applicability of the results. Although all participants were screened for developmental delays, we did not specifically assess the relationship between the degree of developmental delay and OS biomarkers within the ASD group. This may be considered a limitation, and future studies should explore the potential influence of developmental level on redox balance. Furthermore, due to ethical considerations, OS markers were measured in plasma rather than cerebrospinal fluid, which makes it difficult to determine whether peripheral biomarker changes reflect central nervous system processes. While all samples were stored at − 80 °C to preserve biomarker stability, we acknowledge that storage duration, even under optimal conditions, may still have subtle effects on OS levels.

Future studies using population-based samples, including heterogeneous ASD populations across broader age groups, and incorporating additional oxidant/antioxidant markers are needed to better understand the causal relationship between OS and ASD, enhance the generalizability of findings, and evaluate the impact of ASD interventions on redox homeostasis. Longitudinal studies with larger sample sizes are also essential to investigate the temporal dynamics of OS, its role in the progression of ASD, and its interaction with genetic and environmental factors. Finally, a personalized approach that considers individual variability in oxidative stress responses may deepen our understanding of OS in the pathophysiology of ASD. One of the main strengths of our study was its careful design, which included synchronized blood sampling for both cases and controls, the selection of a non-autistic control group, matched for age and gender, and the recruitment of a sufficient number of participants based on a priori power analysis. Rigorous control for potential confounders enhanced the reliability and validity of our results. Moreover, the assessment of interactions between DTDH and other OS parameters, along with the focus on a young age range (2–6 years), distinguishes this study and underscores its significance.

In conclusion, this study enhances the current understanding of the association between ASD and OS, offering a valuable contribution to the literature. For the first time, we comprehensively analyzed DTDH parameters—a relatively novel OS biomarker—alongside classical OS markers (TOS, TAS, OSI, glutathione, and GPx levels) in children with autism. Our findings revealed that these biomarkers exhibited parallel changes. Moreover, we intentionally restricted our sample to a younger age group (2–6 years), allowing for a focused examination of OS dynamics during early developmental stages, which is a critical yet underexplored period in ASD research. However no significant differences were observed between the ASD and control groups across all OS markers in this young cohort. This study underscores the potential impact of age as a critical determinant in investigating the role of OS in autism and highlights the need for further age-stratified research in this field.

## Data Availability

No datasets were generated or analysed during the current study.
